# Monotherapy With Major Antihypertensive Drug Classes and Risk of Hospital Admissions for Mood Disorders

**DOI:** 10.1161/HYPERTENSIONAHA.116.08188

**Published:** 2016-10-12

**Authors:** Angela H. Boal, Daniel J. Smith, Linsay McCallum, Scott Muir, Rhian M. Touyz, Anna F. Dominiczak, Sandosh Padmanabhan

**Affiliations:** From the Institute of Cardiovascular and Medical Sciences (A.H.B., L.M., S.M., R.M.T., A.F.D., S.P.) and Institute of Health and Wellbeing (D.J.S.), University of Glasgow, United Kingdom.

**Keywords:** angiotensin-converting enzyme inhibitors, bipolar disorder, calcium channel blockers, depression, hypertension

## Abstract

Major depressive and bipolar disorders predispose to atherosclerosis, and there is accruing data from animal model, epidemiological, and genomic studies that commonly used antihypertensive drugs may have a role in the pathogenesis or course of mood disorders. In this study, we propose to determine whether antihypertensive drugs have an impact on mood disorders through the analysis of patients on monotherapy with different classes of antihypertensive drugs from a large hospital database of 525 046 patients with follow-up for 5 years. There were 144 066 eligible patients fulfilling the inclusion criteria: age 40 to 80 years old at time of antihypertensive prescription and medication exposure >90 days. The burden of comorbidity assessed by Charlson and Elixhauser scores showed an independent linear association with mood disorder diagnosis. The median time to hospital admission with mood disorder was 847 days for the 299 admissions (641 685 person-years of follow-up). Patients on angiotensin-converting enzyme inhibitors or angiotensin receptor blockers had the lowest risk for mood disorder admissions, and compared with this group, those on β-blockers (hazard ratio=2.11; [95% confidence interval, 1.12–3.98]; *P*=0.02) and calcium antagonists (2.28 [95% confidence interval, 1.13–4.58]; *P*=0.02) showed higher risk, whereas those on no antihypertensives (1.63 [95% confidence interval, 0.94–2.82]; *P*=0.08) and thiazide diuretics (1.56 [95% confidence interval, 0.65–3.73]; *P*=0.32) showed no significant difference. Overall, our exploratory findings suggest possible differential effects of antihypertensive medications on mood that merits further study: calcium antagonists and β-blockers may be associated with increased risk, whereas angiotensin-converting enzyme inhibitors and angiotensin receptor blockers may be associated with a decreased risk of mood disorders.

## Abstract 2

Supplemental Digital Content is available in the text.

Depression and cardiovascular disease are both common disorders and major contributors to the global burden of disease. A bidirectional relationship between depression and cardiovascular disease is thought to exist mainly because of the overlapping pathophysiological processes that underlie both conditions.^1,2^ Bipolar disorder (BD) is associated with a 1.5- to 2.5-fold increased risk of cardiovascular mortality and hypertension,^3^ whereas major depressive disorder (MDD) has a 1.3-fold increased risk of hypertension.^4^ There is accruing data from animal model, epidemiological, and genomic studies that pathways and molecular targets of commonly used antihypertensive drugs may have a role in the pathogenesis or course of mood disorders. Genome-wide association studies support an association of *CACNA1C* polymorphism with BD^5–8^ and unipolar depression,^5,9^ implicating dysfunction of L-type calcium channels in neuropsychiatric disorders. Because L-type calcium channels are the target of the commonly used dihydropyridine (DHP) calcium channel blockers (CCB) commonly used to treat hypertension, there may be potential implications in prescribing these drugs in hypertensive patients who may have an underlying mood disorder. There is also evidence that the brain renin–angiotensin system is involved in proinflammatory mechanisms that mainly affect regions responsible for emotion, which is implicated in mood states of BDs.^10,11^ However, epidemiological evidence for an association between any antihypertensive drug and neuropsychiatric consequences is inconclusive, and it is unclear whether this relationship is because of hypertension per se, its treatment, or both.^12–14^ In this study, we propose to determine whether antihypertensive drugs have an impact on mood disorders through the analysis of patients on monotherapy with different classes of antihypertensive drugs from a large hospital database of 525 046 patients with follow-up for 5 years.

## Methods

### Study Setting and Study Population

The study was conducted on anonymized administrative data from 2 large secondary care hospitals (Western Infirmary and Gartnavel General Hospitals) in the West of Scotland obtained from the National Health Service (NHS) Information and Statistics Division (ISD).^15^ These anonymized data are approved for research by the NHS ISD committee, and the use of the data was reviewed and approved by the Caldicott Guardian (NHS person responsible for protecting the confidentiality of patient and service-user information and enabling appropriate information sharing). The ISD of the NHS in Scotland collects data on all discharges from NHS hospitals using the Scottish Morbidity Record scheme. In Scotland, primary and secondary health care is provided to all citizens, free at point of access, by the NHS. NHS hospitals deliver virtually all elective and emergency hospital care. Data from patient case records are used to code ≤6 diagnoses at the time of discharge according to the World Health Organization Classification of Diseases (ICD-9 before 1996 and ICD-10 after 1996). The database contains hospital admissions and mortality data on 525 046 patients admitted at least once between 1980 and March 2013. Pharmacy refill prescriptions were available from January 2004 onward. The main inclusion criteria were age 40 to 80 years at prescription start date with a medication duration of >90 days. Four mutually exclusive groups based on antihypertensive monotherapy were selected: angiotensin-converting enzyme inhibitors (ACEi) and angiotensin receptor blockers (ARB) grouped as angiotensin antagonists (AA), β-blockers (BB), CCB, and thiazide diuretics (TZ), and a fifth no-antihypertensive therapy (NoAntiHTN) group who were not exposed to any of these 4 antihypertensive drug classes during the study period. A new prescription was defined if the drug was dispensed with at least 3 months of nonreceipt of the drug beforehand.

### Mood Disorder and Comorbidity Coding

Mental health hospital admissions were available from 1980 to March 2013. The diagnoses from the patients’ admissions were available from ISD coding using ICD-9 and ICD-10 codes. We analyzed hospital admissions for major depressive disorders and BDs, and these were defined using the ICD-10 classification system. Using ICD-10 classification system, a diagnosis of major depression requires symptoms to be present >2 weeks and must include 2 key symptoms of low mood, anhedonia, or fatigue along with at least 2 other core symptoms. The symptoms of BDs vary between patients, but classically patients experience periods of prolonged depression alternating with manic episodes. ICD-10 F30-39 codes encompassing mood-affective disorder admissions were selected, and ICD-9 codes were mapped to these to ensure we included all mood disorder admissions (please see Table S1 in the online-only Data Supplement for full coding information). Both the primary and the secondary diagnoses recorded for each hospital admission were included for analysis. Comorbidities at baseline for each subject were determined using 2 indices—Charlson (CCI) and Elixhauser comorbidity index (ECI) scores. These were calculated using the enhanced ICD-9 codes and ICD-10 codes as described in the study by Quan et al.^16^ Because depression is included in Elixhauser index, we repeated the analysis using a modified ECI (mECI) score, which excluded depression in the scoring. All scores were grouped into 3 categories 0, 1, and >1 for analysis.

### Statistical Analysis

Continuous variables were examined using independent *t* tests and 1-way ANOVA and are shown as mean (SD). Categorical variables were compared between groups using χ^2^ tests of association and trend where appropriate and are shown as counts and percentages. Significance was set at *P*<0.05.

Multivariable adjusted binary logistic regression was used to determine predictors of mood disorder. Regression models were constructed adjusting for age and sex in all models and adding each comorbidity score (CCI, ECI, and mECI) separately in different models. Cox proportional hazards models were used to determine the risk of incident hospital admission for mood disorder in the antihypertensive monotherapy and NoAntiHTN groups during a 5-year follow-up. The baseline was the date of first prescription of the antihypertensive drug and April 1, 2004, for the NoAntiHTN group. Model 1 was adjusted for age at baseline and sex, and then, the 3 comorbidity scores were then added individually to this original model (models 2–4). Fulfillment of proportionality assumptions were checked by inspecting log minus log plots. The referent group was AA for each model. We also conducted a sensitivity analysis for MDD only. All analyses were performed using SPSS version 20.0.0 (IBM Corp) and R version 3.2.0 (The R Foundation for Statistical Computing).

## Results

### Demographic and Clinical Characteristics

After all exclusions, there were 144 066 eligible individuals; the study flow chart is presented in Figure. The mean age was 55.5 years, and 52% were female patients, and there were 299 incident admissions with mood disorders (84% MDD, 15% BD, and 1% manic episode, persistent or unspecified mood disorder), and of these, 251 were because of MDD (Table 2). There were 18 799 deaths during the 5-year follow-up period. There were 111 936 NoAntiHTN subjects and 32 130 patients on monotherapy (AA 33.7%; BB 36.1%; CCB 18.3%; and TZ 11.9%). More than half the patients had a CCI, ECI, and mECI score of 0 (61.9%, 53.5%, and 53.8%, respectively).

**Table 1.**
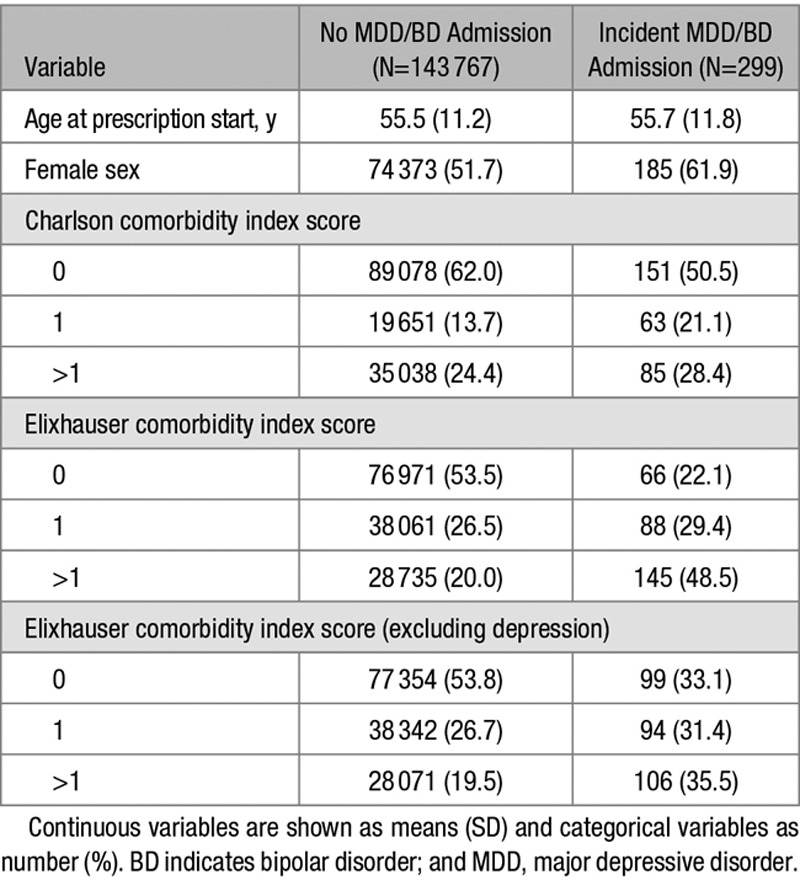
Baseline Population Characteristics Stratified by Hospital Admission Status

**Table 2.**
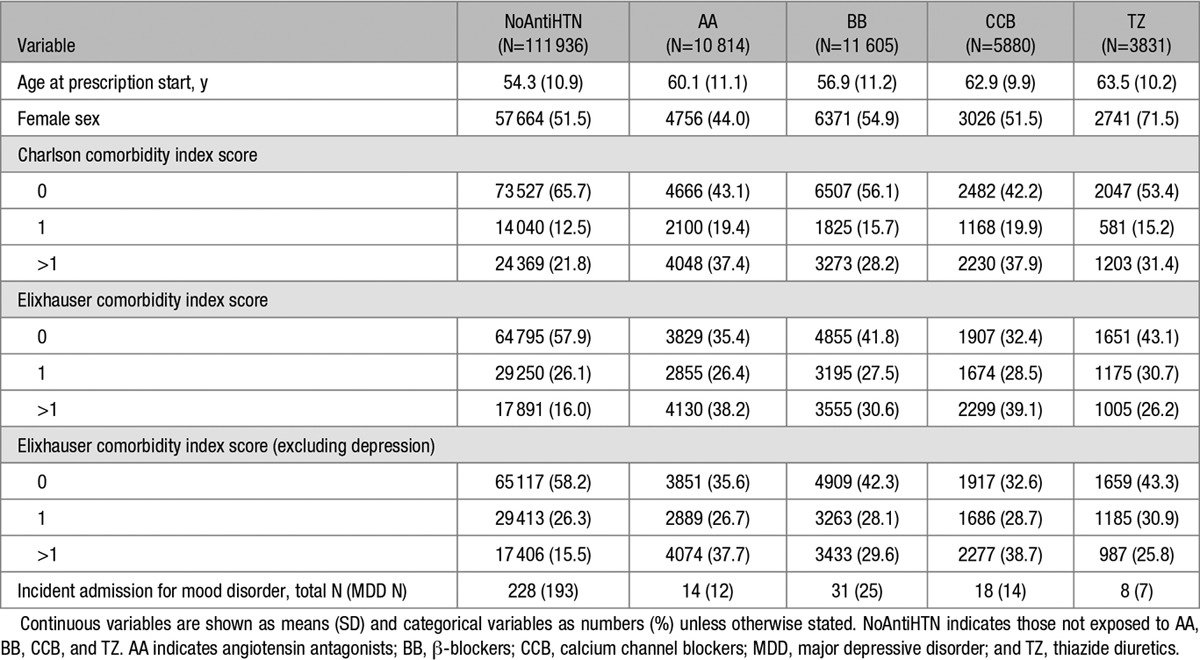
Baseline Population Characteristics Stratified by Monotherapy Drug Regimen

**Figure.**
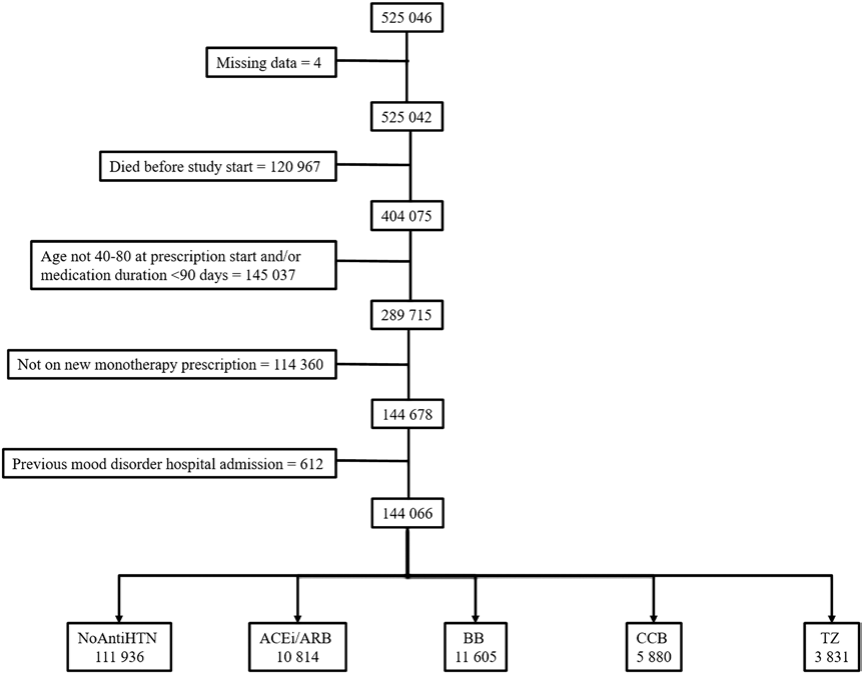
Study flowchart. NoAntiHTN indicates those not exposed to angiotensin antagonists (AA), β-blockers (BB), calcium channel blockers (CCB), and thiazide diuretics (TZ). ACEi indicates angiotensin-converting enzyme inhibitors; and ARB, angiotensin receptor blocker.

Comparing the groups with and without an incident admission with mood disorder, the group with mood disorder admissions were predominantly women (62%) and had a higher burden of comorbidities at baseline (Table 1).

Compared with the NoAntiHTN group, the antihypertensive monotherapy groups were older and also had a higher burden of comorbidities (Table 2). The AA group had a higher proportion of men (56%), whereas TZ was predominantly of women (71.5%).

Multivariable adjusted binary logistic regression analysis showed a linear increase in odds of mood disorder hospital admissions with Elixhauser score irrespective of the inclusion of depression in the calculation of the score and female patients had a 1.5-fold increased odds (Hosmer–Lemeshow goodness-of-fit *P*>0.05 for ECI and mECI models, C statistic=0.584 [95% confidence interval: 0.551–0.617]; please see Table 3 for ECI and Tables S2 and S3 for CCI and mECI models).

**Table 3.**
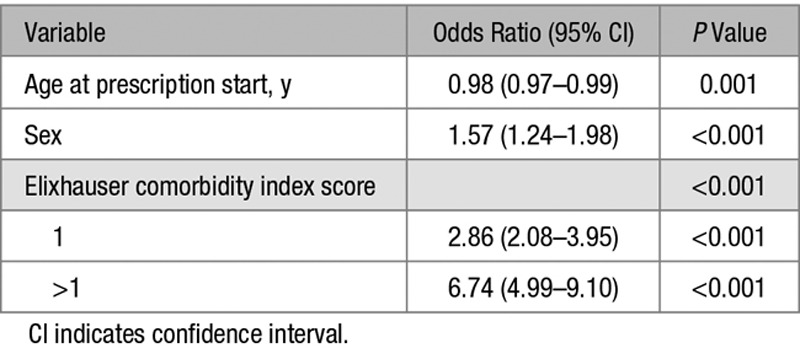
Binary Logistic Regression Model for Age, Sex, and Elixhauser Comorbidity Index Score

### Antihypertensive Drugs and Risk of Mood Disorder Admission

The median time to mood disorder hospital admission was 847 days for the 299 admissions (641 684 person-years of follow-up). Kaplan–Meier analysis showed that the CCB group is associated with the highest risk of mood disorder admissions and AA with the lowest (log-rank *P*=0.006). The median time to admission for TZ, BB, NoAntiHTN, CCB, and AA were 436.5, 451, 710.5, 744.5, and 933.5 days, respectively. Kaplan–Meier analysis showed a significant difference in the survival times between groups (Mantel-Cox log-rank test *P*=0.006).

The results of the multivariable adjusted Cox proportional hazards models are presented in Table 4 and in Figure S1 AA group was associated with the lowest risk of mood disorder admissions. Compared with the AA group, the CCB and BB groups were associated with a 2-fold increased risk of mood disorder admissions. Interestingly, compared with the NoAntiHTN group, AA group showed a 53% decreased risk of mood disorder admissions, suggesting a possible protective role. The risk associated with TZ did not attain statistical significance in any of the models.

**Table 4.**
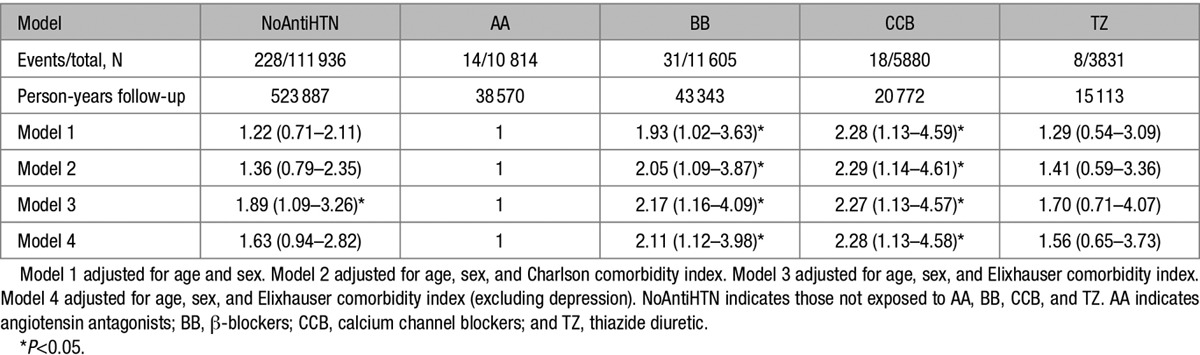
Cox Proportional Hazards Model Results for Risk of Mood Disorder Hospital Admission and Different Antihypertensives Drug Classes

### Sensitivity Analysis

We repeated the analysis for MDD admissions only. Multivariable binary logistic regression analysis showed a similar linear increased odds of mood disorder admissions with higher levels of the Elixhauser scores, and female patients had a 1.7 increased odds (Tables S4 and S5). In all Cox proportional hazards models (Table S6), the AA group was again associated with the lowest risk of admission and CCB, and BB and NoAntiHTN groups were associated with 2-fold increased risks of events, although CCB did not reach statistical significance in these models.

## Discussion

In our exploratory large cohort study, we show that antihypertensive drug classes may have different effects on risk of hospital admissions with mood disorder diagnosis. An increased risk of mood disorder admissions was associated with BB and CCB therapy. Interestingly, we found that ACEi/ARB therapy had a neutral effect (or reduced risk) on mood disorders because this group showed the lowest risk of admissions across all groups including the group that received no antihypertensive drugs. We also show that the presence of comorbidities significantly increased the risk of mood disorders in the 5-year follow-up period, and this is in line with literature indicating individuals with serious mental illnesses have an increased number/risk of comorbidities.^17–20^ Our finding that female patients have an increased risk of mood disorder admissions is not novel. It is well established that female patients have a 2-fold increased risk of depression^21^ and possibly also a higher risk of BD.^22^

Multiple lines of evidence suggest a role for CCB in mental disorders, but no conclusive results have been drawn thus far. Mouse models of CCB therapy have shown variable results, but evidence from these models should be approached carefully because of differences between species such as transport across the blood–brain barrier.^12^ DHP CCB in mouse models of depression have shown promising evidence of antidepressant effects, with antidepressants facilitating this effect, but non-DHP CCBs have been shown to lack activity or produce the opposite effect.^12^ Despite this promising evidence, most clinical trials have focused on manic phase or rapid cycling BD.^12^ Clinical studies have been underpowered with heterogeneous designs,^12^ but they have found preliminary positive findings with symptom improvement with DHP in BD^12,23^ and depression secondary to cerebrovascular disease.^24^ For example, a recent pilot clinical trial by Ostacher et al^23^ of isradipine in 10 normotensive BD patients showed a promising reduction in depressive symptoms with no significant adverse effects despite a high drop-out rate. We studied 144 066 individuals and found that CCB increased the risk of mood disorder hospital admission 2-fold in comparison to ACEi/ARB. Our study expands on previous research and shows a negative effect of CCB in mood disorders in comparison to previous clinical trials. However, in our large cohort on monotherapy prescription, we investigated incident hospital admissions in comparison to changes in symptoms. We also investigated all classes of CCB, and as such, we cannot comment on DHP specifically, but further research could examine the relationship between DHP and non-DHP CCBs and hospital admissions. It is important to study CCB in neuropsychiatric disorders as genome-wide association studies have implicated the dysfunction of L-type calcium channels in both BD^5–8^ and unipolar depression,^5,9^ but the direction of this effect is still in question. In addition, there are conflicting results of verapamil and its possible antimanic efficacy, but a recent clinical trial showed that its combination with lithium was highly efficacious, whereas monotherapy was not. The authors hypothesized that this effect may be because of the additive attenuation of protein kinase C.^25^ Overall, although we showed a negative effect of CCB leading to an increase in incident hospital admissions, further investigations are required.

We showed BB to be associated with an increase in mood disorder admission, which aligns well with depression being listed as an uncommon side effect of this drug class. However, a recent large systematic review in 2002 showed that BB therapy was not associated with a significant increase in reporting depressive symptoms,^26^ but we investigated severe mood disorders requiring hospitalization and not symptom reporting. Furthermore, previous research from the 20th century found that although propranolol use was variably associated with depression, results from observational studies showed conflicting results. For example, one study looking at the incidence of depression with a new antihypertensive prescription found no additional risk with BB compared with other antihypertensives; however, there were limitations such as only achieving modest statistical power.^27^ Thiessen et al^28^ showed that patients newly commenced on BB had a higher rate of antidepressant prescriptions compared with diuretics. In contrast to these studies, our study looked at the more severe end of the spectrum of mood disorders requiring hospital admissions where we show a clear increase in risk between with BB and CCB. Autonomic dysfunction is a proposed mechanism for both BD and MDD, and this is reflected by patients having higher heart rates and lower heart rate variability, which is known to lead to an increased cardiovascular disease risk.^29^ Recently, Taylor^29^ put forth a suggestion that BB could be considered in depression on a case-by-case basis as they reduce heart rates while increasing heart rate variability.

Of greater interest is the lower risk for mood disorders seen in those on ACEi/ARB monotherapy in our study. People with a history of depression have been reported to be at an increased risk of dementia,^30^ and interestingly, antihypertensives targeting the renin–angiotensin system have recently shown a possible reduction in the incidence and progression of Alzheimer disease.^31^ A recent systematic review including >1 million individuals found that all antihypertensive drugs had benefits on overall cognition, with ARB being more effective than ACEi.^32^

TZ showed a nonsignificant increased risk of mood disorder admission in comparison to ACEi/ARB, and this may reflect the low number of events in this group. Further studies with larger sample sizes are required to establish a detrimental effect of TZ on mood disorders. It is known that TZ minimally cross the blood–brain barrier but can affect sodium and calcium levels, resulting in psychiatric complications.^13^

Several interacting biological systems might contribute to shared pathophysiological mechanisms between mood disorders and cardiovascular disease. These include overactivity of the hypothalamic–pituitary–adrenal axis,^1,33,34^ neuroinflammation, oxidative stress,^1^ and endothelial dysfunction.^1,35^ Some of these systems, particularly the hypothalamic–pituitary–adrenal axis, may be influenced by antihypertensive medications. There is now evidence that the renin–angiotensin system, especially in the brain, plays an important role in cognition, depression, and behavior and that inhibition of the renin–angiotensin system may have therapeutic potential in mood disorders.^36,37^

The strengths of this study include the observation of a large cohort with longitudinal follow-up data; the ability to study groups on monotherapy with different antihypertensive drugs and a large NoAntiHTN group; the availability of hospital morbidity data for at least 20 years preceding the study start date, enabling an accurate calculation of comorbidity scores; and the availability of refill prescription data, which is a better marker of patient receipt of the drugs. Our study has important limitations. We only investigated the incidence of severe mood disorders coded on hospital discharge data, and thus, our results do not include milder levels of mood disorders that are treated in the community. The results of our study may be the result of confounding through unmeasured covariates, and further validation in independent studies is required. The absolute risks for admissions with mood disorders are small, and future prospective studies should incorporate more granular measures of neuropsychiatric evaluation for assessment of outcomes. Our population cohort, although large, is derived from 2 large secondary and tertiary care hospitals, and hence, the generalizability of our findings is unknown, and further studies are needed to validate our results. The global prevalence estimates from the WHO for depression and BD are 350 million and 60 million, respectively^38^; thus, it is not surprising that >77% of incident admissions in each monotherapy group are because of MDD. Through this and our sensitivity analysis, we expect that most of our results are driven by MDD, but an effect of CCBs on BD outcomes cannot be discounted as we are underpowered to comment on this, but we show a direct effect of MDD admissions. Adherence and exposure to the drugs and ethnicity of individuals are unknown.

## Perspectives

Mental health is an under-recognized area in hypertension clinic practice, and our study highlights the importance of reviewing mental health diagnoses and progression. Until validated, it is worthwhile remembering that antihypertensive drugs may have an impact on mental health. The neutral effect (or reduced risk) exerted by ACEi/ARB could lead to consideration of these drugs in certain subgroups of patients at risk of mood disorders.

## Sources of Funding

S. Padmanabhan is funded by the Medical Research Council (MR/M016560/1 and The AIM-HY Study) and the British Heart Foundation (PG/12/85/29925 and CS/16/1/31878). D.J. Smith is funded by a Lister Institute Prize Fellowship. L. McCallum is funded by a BHF fellowship (FS/14/52/30901). A.F. Dominiczak has funding from the Scottish Ecosystem for Precision Medicine.

## Acknowledgments

We would like to thank the Information and Statistics Division, NHS Scotland, for access to the database.

## Disclosures

None.

## Supplementary Material

## Novelty and Significance

### What Is New?

Antihypertensive drug classes have a differential effect on hospital admissions for mood disorders. β-Blocker and calcium channel blocker therapies seem to be associated with a higher risk for mood disorder hospital admissions. Angiotensin-converting enzyme inhibitors and angiotensin receptor blockers may exert a protective effect compared with other antihypertensive drugs and no exposure.

### What Is Relevant?

Mental health is under-recognized in hypertension clinical practice, and the possible impact antihypertensive drugs has on mental health is important to be aware of.

### Summary

The effect of antihypertensive drug therapy on mental health of patients warrants further study.
