# Supplementary material for: Monotherapy With Major Antihypertensive Drug Classes and Risk of Hospital Admissions for Mood Disorders
Source: Hypertension. 2016 Oct 12;68(5):1132–8. doi: 10.1161/HYPERTENSIONAHA.116.08188 (PMC5058642; doi:10.1161/HYPERTENSIONAHA.116.08188)
Supplement: Supplementary file 1 [file hyp-68-1132-s001.docx]

**SUPPLEMENTARY DATA**

**Monotherapy with major Antihypertensive DRug classes and risk of hospital admissions for mood disorders**

**Angela H Boal^1^, Daniel J Smith^2^, Linsay McCallum^1^, Scott Muir^1^, Rhian M Touyz^1^, Anna F Dominiczak^1^, Sandosh Padmanabhan^1^**

1. University of Glasgow, Institute of Cardiovascular and Medical Sciences, Glasgow, UNITED KINGDOM
2. University of Glasgow, Institute of Health and Wellbeing, Glasgow, UNITED KINGDOM

**Table S1.** Full coding information for mood disorder hospital admissions

| Mental health admission diagnosis | ICD-9 codes | ICD-10 codes |
| --- | --- | --- |
| Major depressive disorder | 296.2-296.3, 298.0 | F32-33 |
| Bipolar disorder | 296.4-296.8* | F31 |
| Manic episode | 296.0-296.1 | F30 |
| Persistent mood disorder | 300.4 | F34 |
| Other or unspecified mood disorder | 269.9 | F39 |

*As 296.8 codes encompassed mostly bipolar disorder (BD) codes, it was classified as BD for this study

ICD-9 indicates International Classification of Diseases, 9^th^ revision; ICD-10, International Classification of Diseases, 10^th^ revision

**Table S2.** Binary logistic regression model for age, sex and Charlson comorbidity score

| Variable | Odds ratio [95% confidence interval] | *P* |
| --- | --- | --- |
| Age at prescription start | 1.00 [0.986-1.007] | 0.516 |
| Sex | 1.54 [1.22-1.94] | <0.001 |
| Charlson comorbidity index score |  | <0.001 |
| 1 | 1.96 [1.46-2.64] | <0.001 |
| >1 | 1.46 [1.11-1.92] | 0.007 |

**Table S3.** Binary logistic regression model for age, sex and Elixhauser comorbidity index score (excluding depression)

| Variable | Odds ratio [95% confidence interval] | *P* |
| --- | --- | --- |
| Age at prescription start | 0.99 [0.98-1.00] | 0.052 |
| Sex | 1.55 [1.23-1.96] | <0.001 |
| Elixhauser comorbidity index score (excluding depression) |  | <0.001 |
| 1 | 1.99 [1.50-2.65] | <0.001 |
| >1 | 3.23 [2.43-4.30] | <0.001 |

**Table S4.** Binary logistic regression model for age, sex and Elixhauser comorbidity index score for sensitivity analysis of major depressive disorder

| Variable | Odds ratio [95% confidence interval] | *P* |
| --- | --- | --- |
| Age at prescription start | 0.98 [0.97-0.99] | 0.001 |
| Sex | 1.67 [1.29-2.16] | <0.001 |
| Elixhauser comorbidity index score |  | <0.001 |
| 1 | 2.84 [1.99-4.04] | <0.001 |
| >1 | 7.07 [5.09-9.80] | <0.001 |

**Table S5.** Binary logistic regression model for age, sex and Elixhauser comorbidity index score (excluding depression) for sensitivity analysis of major depressive disorder

| Variable | Odds ratio [95% confidence interval] | *P* |
| --- | --- | --- |
| Age at prescription start | 0.99 [0.98-1.00] | 0.027 |
| Sex | 1.65 [1.28-2.14] | <0.001 |
| Elixhauser comorbidity index score |  | <0.001 |
| 1 | 1.84 [1.35-2.52] | <0.001 |
| >1 | 3.22 [2.37-4.39] | <0.001 |

**Table S6.** Cox Proportional Hazard Model Data for sensitivity analysis

| Model | NoAntiHTN | AA | BB | CCB | TZ |
| --- | --- | --- | --- | --- | --- |
| Events/total N | 193/111936 | 12/10814 | 25/11605 | 14/5880 | 7/3831 |
| Model 1 | 1.19 (0.66-2.14) | 1 | 1.79 (0.90-3.56) | 2.07 (0.96-4.48) | 1.31 (0.51-3.34) |
| Model 2 | 1.36 (0.75-2.45) | 1 | 1.93 (0.97-3.85) | 2.08 (0.96-4.49) | 1.00 (0.99-1.01) |
| Model 3 | 1.86 (1.03-3.35)* | 1 | 2.02 (1.02-4.04)* | 2.06 (0.95-4.45) | 1.73 (0.68-4.41) |
| Model 4 | 1.59 (0.88-2.88) | 1 | 1.97 (0.99-3.92) | 2.06 (0.95-4.47) | 1.59 (0.62-4.04) |

*P<0.05

Model 1 adjusted for age and sex

Model 2 adjusted for age, sex and Charlson comorbidity index

Model 3 adjusted for age, sex and Elixhauser comorbidity index

Model 4 adjusted for age, sex and Elixhauser comorbidity index (excluding depression)

AA indicates angiotensin-converting enzyme inhibitor and angiotensin receptor blockers; BB, beta blockers; CCB, calcium channel blockers; TZ, thiazide diuretics


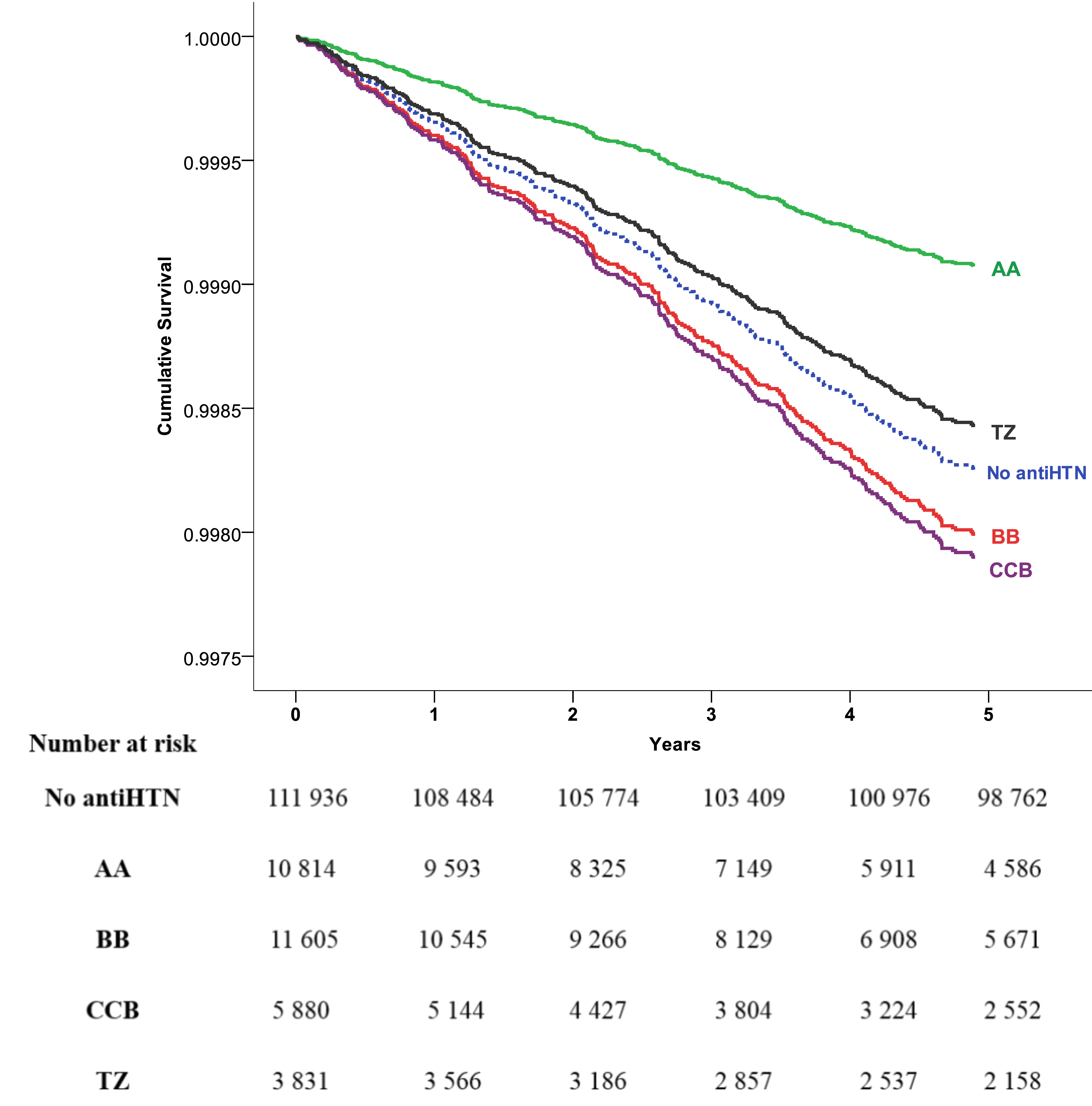


**Figure S1:** Cumulative hazard plot for risk of mood disorder hospital admission and different antihypertensive drug classes

NoAntiHTN indicates those not exposed to AA, BB, CCB, TZ; AA, angiotensin-converting enzyme inhibitors and angiotensin receptor blockers; BB, beta blockers; CCB, calcium channel blockers; TZ, thiazide diuretics
